# Improving the Antimycobacterial Drug Clofazimine through Formation of Organic Salts by Combination with Fluoroquinolones

**DOI:** 10.3390/ijms24021402

**Published:** 2023-01-11

**Authors:** Clara M. Bento, Ana Teresa Silva, Bruno Mansano, Luísa Aguiar, Cátia Teixeira, Maria Salomé Gomes, Paula Gomes, Tânia Silva, Ricardo Ferraz

**Affiliations:** 1 i3S—Instituto de Investigação e Inovação em Saúde, Universidade do Porto, 4200-135 Porto, Portugal; 2 IBMC—Instituto de Biologia Molecular e Celular, Universidade do Porto, 4200-135 Porto, Portugal; 3 Programa Doutoral em Biologia Molecular e Celular (MCBiology), Instituto de Ciências Biomédicas Abel Salazar, Universidade do Porto, 4050-313 Porto, Portugal; 4 LAQV-REQUIMTE, Departamento de Química e Bioquímica, Faculdade de Ciências da Universidade do Porto, 4169-007 Porto, Portugal; 5 ICBAS—Instituto de Ciências Biomédicas Abel Salazar, Universidade do Porto, 4050-313 Porto, Portugal; 6 CISA—Ciências Químicas e das Biomoléculas, Escola Superior de Saúde, Politécnico do Porto, 4200-072 Porto, Portugal

**Keywords:** antibiotics, GUMBOS, ionic liquids, *Mycobacterium*, organic salts, repurposing, rescuing

## Abstract

This work reports the synthesis, structural and thermal analysis, and in vitro evaluation of the antimicrobial activity of two new organic salts (OSs) derived from the antimycobacterial drug clofazimine and the fluoroquinolones ofloxacin or norfloxacin. Organic salts derived from active pharmaceutical ingredients (API-OSs), as those herein disclosed, hold promise as cost-effective formulations with improved features over their parent drugs, thus enabling the mitigation of some of their shortcomings. For instance, in the specific case of clofazimine, its poor solubility severely limits its bioavailability. As compared to clofazimine, the clofazimine-derived OSs now reported have improved solubility and thermostability, without any major deleterious effects on the drug’s bioactivity profile.

## 1. Introduction

The genus *Mycobacterium* comprises more than 200 species of bacteria, some of them responsible for pulmonary and extrapulmonary diseases in humans and other animals. Mostly found in soil or water sources, these pathogenic bacteria can be separated into two categories: the species of the *M. tuberculosis* complex and *M. leprae*, agents of tuberculosis and leprosy, respectively, and non-tuberculous mycobacteria (NTM), such as the species from the *M. avium* complex (MAC) [1]. The prevalence of disease caused by NTM infection has been increasing globally in the last decades, in opposition to what is observed for tuberculosis [2]. Even though they predominantly affect patients who are immunocompromised or with a history of pulmonary disease, NTM-positive cultures, especially of MAC, are frequently obtained from patients without any of the known risk factors [3]. In the host, mycobacteria infect phagocytic cells, such as macrophages, in which the bacteria replicate inside small vacuoles by inhibiting the phagosome–lysosome fusion [4]. The basis of MAC disease therapy is a macrolide drug, typically azithromycin or clarithromycin, which should be combined with ethambutol and a rifamycin to avoid macrolide resistance. A fourth drug is added to the regimen in more severe cases [5,6]. The treatment is long and often results in discontinuation or modification due to toxicity. Moreover, many antimycobacterial drugs have their therapeutic efficacy hampered by quite poor solubility or low oral bioavailability. On the other hand, developing new drugs from scratch is an unsustainable, time-consuming, and expensive process, which does not guarantee that the candidate with the best therapeutic index will not induce the selection of resistant strains. This underpins the wide interest in rescuing and repurposing known active pharmaceutical ingredients (APIs) and in using drug combinations instead of monotherapies, privileging the most simple and cost-effective approaches possible [7].

In connection with the above, the 2007 paper “The third evolution of ionic liquids: active pharmaceutical ingredients” by Hough et al. [8] turned the spotlight onto the paradigm shift that has been occurring over the past decade regarding ionic liquids (ILs): the growing relevance of ILs in drug discovery and formulation, including the development of ILs derived from APIs (API-ILs) as a cost-effective way to rescue or repurpose the latter [9,10,11,12]. In fact, the conversion of APIs into API-ILs may help to overcome some issues such as poor solubility, polymorphic conversion, or low bioavailability, taking advantage of the unique properties of ILs [13]. While ILs are commonly defined as organic salts (OSs) with a melting point below 100 °C [12,13], which includes room-temperature ionic liquids (RTILs) [14], this is a somewhat limiting definition. It is the type of interactions established by and within such OSs, rather than their melting points, which differentiates them from the classic inorganic salts [15]. Weaker Coulombic interactions and lower cohesive energies in the solid phase, due to a lack of ion symmetry and low charge density, confer to OSs unique properties, such as their much lower melting points compared to conventional salts and higher solubility compared to their parent organic building blocks [13]. A recent trend to classify this special group of OSs, irrespective of their melting points, is to gather them in the so-called Group of Uniform Materials Based on Organic Salts (GUMBOS) [16,17,18]. Regardless of which may be the most adequate terminology, pairing APIs with selected organic ions offers the possibility to fine-tune the biological and physicochemical properties of the resulting API-derived organic salts (API-OSs) [15,19]. Hence, the production of API-OSs emerges as an attractive way to improve the therapeutic index of known APIs whose clinical use may be hampered by bioavailability and/or solubility issues [12,20,21,22,23,24,25].

**Figure 1:**
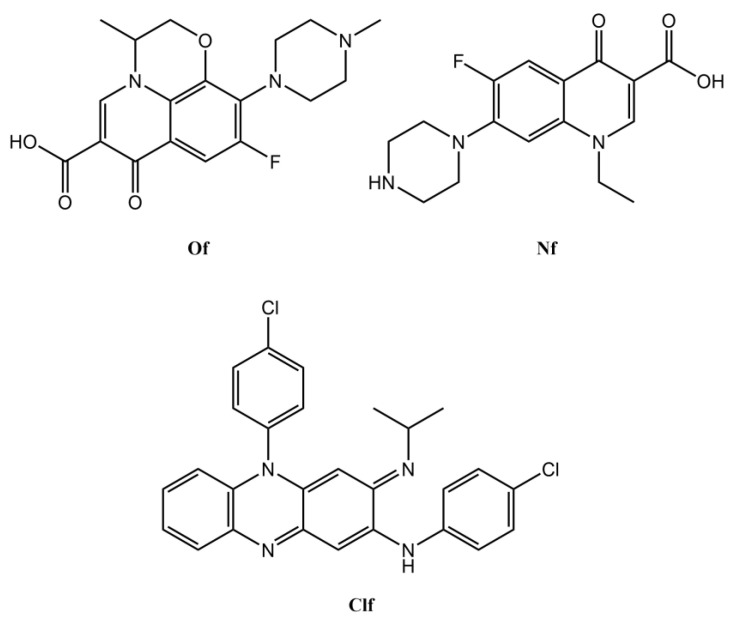
Structural formulae of the fluoroquinolones ofloxacin (Of) and norfloxacin (Nf), and of the antimycobacterial drug clofazimine (Clf).

Bearing all the above in mind, we have recently considered the possibility of improving the solubility of the antimycobacterial drug clofazimine (Clf), while decreasing the chance of selecting clofazimine-resistant strains, by producing two novel clofazimine-derived OSs via the acid–base pairing of that drug with two fluoroquinolones, namely, ofloxacin (Of) or norfloxacin (Nf), as shown in Figure 1. Fluoroquinolones inhibit bacterial DNA gyrase and topoisomerase IV, two key enzymes involved in DNA synthesis [26]. Relevantly, some fluoroquinolones have shown activity against extracellular and intracellular *M. avium* in vitro and in vivo in infected mice [27]. In MAC patients, the addition of fluoroquinolones to the recommended three-drug therapy results in similar treatment outcomes but implies more adverse side effects [28]. Clf is part of the standard treatment against leprosy, although it was deemed ineffective in monotherapy against *M. tuberculosis* [29]. Retrospective and observational studies show that Clf is active against NTM, especially *M. avium* [30,31,32], being currently in a Phase II clinical trial for the treatment of pulmonary *M. avium* disease (https://clinicaltrials.gov/ct2/show/NCT02968212, accessed on 4 January 2023). Its mechanism of action is not completely clear and is thought to be multifactorial, acting in the respiratory chain and ion transporters of mycobacteria, promoting the release of reactive oxygen species (ROS) and binding to DNA, disturbing it [29]. Although Clf affects the host’s innate response by inducing apoptosis in macrophages [33], its ability to increase the humoral immune response [34] is an advantage in the treatment of immunocompromised NTM patients, such as those infected with HIV [35].

## 2. Results and Discussion

### 2.1. Synthesis and Physico-Chemical Properties of the Clf-Derived OSs

The Clf-derived OSs were produced by combination with either Of or Nf, through the acid-base neutralization method [21,22], using methanol/dichloromethane as the solvent system, as given in detail in Section 3 (Materials and Methods). The target API-OSs, [Clf][Of] and [Clf][Nf], were obtained in nearly quantitative (100%) yields as red powders, hence precluding their classification as RTILs. Spectroscopic data (see Materials and Methods) agreed with their expected structures.

**Figure 2:**
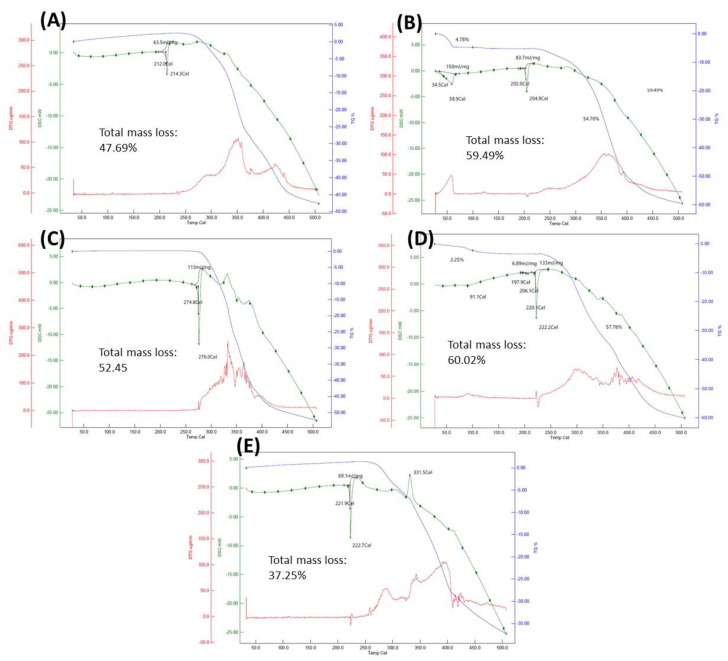
Thermograms obtained by STA of the API-OSs and their parent APIs: (**A**) [Clf][Of]; (**B**) [Clf][Nf]; (**C**) Of; (**D**) Nf; (**E**) Clf. Green trace—differential scanning calorimetry (DSC); blue trace—thermogravimetric analysis (TG); red trace—1st order derivative of the blue trace (DTG).

Considering that thermal stability is an important issue for any API or derived formulation, both API-OSs were next tested by simultaneous thermal analysis (STA) which combines thermogravimetry with differential scanning calorimetry. Thermograms were obtained for the API-OSs and their parent fluoroquinolones (Figure 2), providing their respective melting points and decomposition temperatures. As shown in both Figure 2 and Table 1, the two new API-OSs have melting points lower than those of their respective parent APIs, as expected, but in all cases well above 100 °C, which is generally acknowledged as the threshold temperature separating common ILs from other types of GUMBOS. Additionally, all the compounds exhibited one thermal decomposition event, which occurs at higher temperatures for the API-OSs compared to Clf alone. Therefore, the thermostability of this parent antimycobacterial drug is improved when it is forming an ionic pair with the selected fluoroquinolones.

Data obtained (Table 1) also show a clear improvement in the solubility of Clf upon its ionic pairing with either of the two fluoroquinolones used. Of note, both this solubility study, as well as in vitro bioactivity data (see Section 2.2) were obtained from stock solutions of all compounds in dimethylsulfoxide (DMSO) as an alternative to water, where Clf is virtually insoluble [36]. DMSO has been previously established as an adequate solvent for studies focused on Clf, including susceptibility testing on *Mycobacteria* [37]. Hence, the maximum concentration reached for soluble Clf was 10.2 mM, whereas 15.8 and 17.3 mM could be attained for the [Clf][Of] and the [Clf][Nf] OSs, corresponding to a solubility improvement of ca. 57 and 72%, respectively, as compared to the parent antimycobacterial drug. Any gain in the solubility of the highly lipophilic Clf in polar solvents is important and prospectively of clinical relevance in the long run: Clf is classified as a class II drug in the Biopharmaceutics Classification System due to its poor solubility in aqueous/polar media, including gastro-intestinal luminal fluids, thus implying a quite limited oral bioavailability [38,39]. Indeed, Clf absorption rates in humans vary from 45 to 62% and the drug tends to be deposited predominantly in fatty tissue, whereas going above the optimal human daily dosage (50–100 mg) to overcome oral bioavailability limitations is not an option due to toxicity issues [36].

### 2.2. In Vitro Activity of the Clf-Derived OSs against M. avium

**Figure 3:**
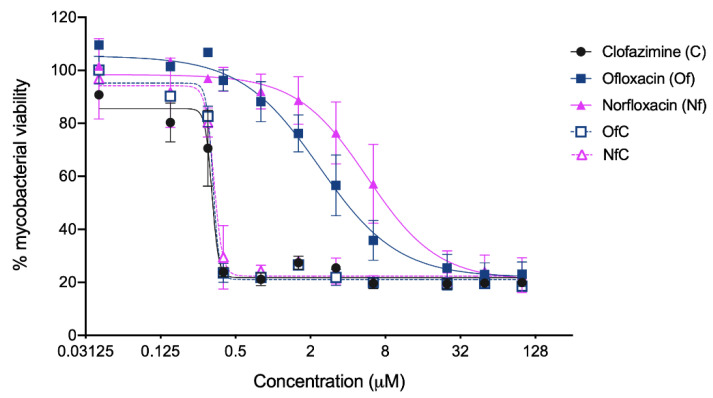
Antimycobacterial activity of the API-OSs and their parent drugs. The symbols represent the averages ± standard deviations of two to three independent experiments, presented as percentages of viable mycobacteria relative to the non-treated mycobacteria. The lines represent the non-linear 4PL regression obtained in the GraphPad software for each experimental condition.

**Figure 4:**
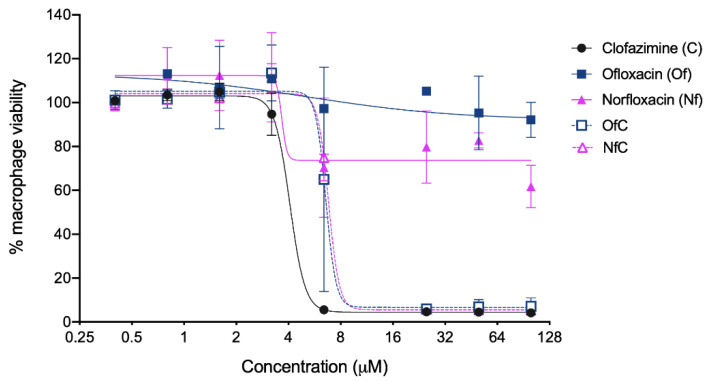
Toxicity of the Clf-derived OSs and their parent drugs to host mammalian cells (murine BMM). The symbols represent the averages ± standard deviations of two to three independent experiments, presented as percentages of viable macrophages relative to non-treated macrophages. The lines represent the non-linear 4PL regression obtained in the GraphPad software for each experimental condition.

**Figure 5:**
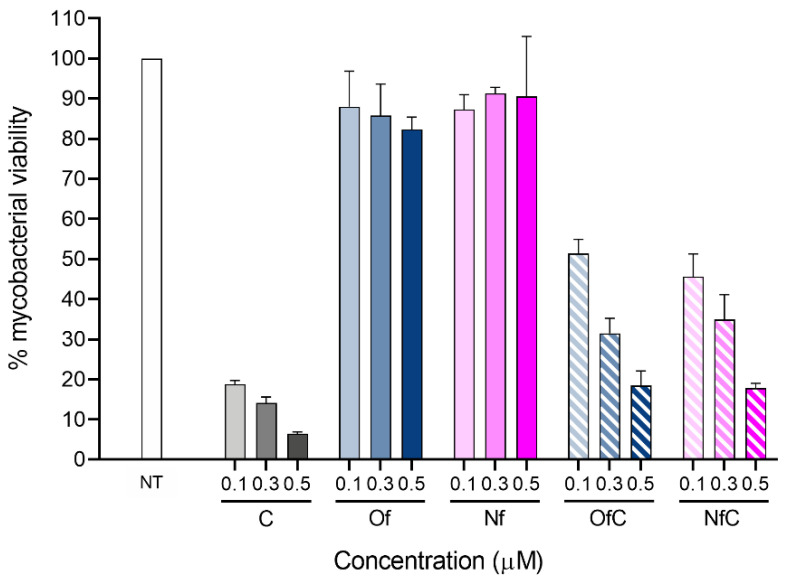
Activity of the API-OSs and respective parent drugs against intracellular mycobacteria. The graph shows the averages + standard deviations of one representative experiment, presented as the percentage of bacterial load to nontreated macrophages on day 5 of incubation.

The antimycobacterial activity and toxicity to host cells of both Clf-derived OSs were tested in vitro, and the obtained results are shown in Table 1 and Figure 3, Figure 4 and Figure 5.

The susceptibility of *M. avium* to the API-OSs and their parent drugs was first assessed in axenic culture to determine the IC_50_ of each test compound. To this end, the bacteria were incubated with increasing concentrations of the compounds for 7 days at 37 °C and their viability was assessed by the resazurin assay. Both API-OSs were found to be equipotent to Clf and more potent than each of the individual fluoroquinolones (Table 1, Figure 3).

Considering that, after invading its mammalian host, *M. avium* replicates mainly inside macrophages, the toxicity of test compounds to infected murine bone marrow-derived macrophages (BMM) was also assessed. This was carried out by treating BMM infected with *M. avium* 2447 SmT with increasing concentrations of the test compounds for 5 days at 37 °C and 7% CO_2_. Macrophage viability was assessed through a resazurin reduction assay. As shown in Table 1 and Figure 4, though being more toxic to mammalian cells than their parent fluoroquinolones, the Clf-derived OSs seem less toxic to BMM than Clf itself. We are aware that the confidence intervals for LD_50_ values shown in Table 1 for Clf and its derived OSs have some degree of overlap which, alongside the large standard deviations depicted on the macrophage viability curves shown in Figure 4, are not statistically significant different. Still, visual inspection of the viability curves in Figure 4 does suggest a decrease in toxicity for the OSs as compared to the parent Clf, and the same is hinted by the LC_50_ confidence intervals in Table 1.

Then, to test the activity of the compounds against intracellular mycobacteria, BMM infected with *M. avium* 2447 SmT were treated with three concentrations of the test compounds that were not toxic to the macrophages (0.1, 0.3 and 0.5 μM) for 5 days at 37 °C and 7% CO_2_. The intracellular bacterial loads were quantified by the colony-forming units (CFUs) assay (Figure 5). Relevantly, even though the Clf-derived OSs were less active than the parent Clf, they still reduced the bacterial load up to 80% at the tested concentrations, being considerably more active against intracellular *M. avium* than their parent fluoroquinolones.

## 3. Materials and Methods

### 3.1. Compounds

The API-OSs were prepared as follows: a solution of either ofloxacin (Sigma-Aldrich, St. Louis, MO, USA; 36.1 mg; 100 µmol) or norfloxacin (Sigma-Aldrich, St. Louis, MO, USA; 31.9 mg; 100 µmol) in methanol (VWR International, Carnaxide, Portugal; 15 mL) was added dropwise to a solution of clofazimine (Sigma-Aldrich, St. Louis, MO, USA; 47.2 mg; 100 µmol) in dichloromethane (15 mL). The acid–base reaction was allowed to proceed overnight at room temperature, under magnetic stirring and protected from light. Then, the solvents were removed by evaporation under reduced pressure on a rotatory evaporator, and the waxy solids obtained were dried overnight in a vacuum oven at 50 °C. The API-OSs were analyzed by proton (^1^H-) and carbon-13 (^13^C-) nuclear magnetic resonance (NMR) on a Bruker Avance III 400 MHz instrument (Centro de Materiais da Universidade do Porto, Porto, Portugal), as well as by electrospray ionization-ion trap mass spectrometry (ESI-IT MS) on a Finnigan Surveyor LCQ DECA XP MAX spectrometer operating with electrospray ionization and ion trap quadrupole detection (Department of Chemistry and Biochemistry, Faculty of Sciences, University of Porto, Porto, Portugal), as given in detail in the Supplementary Information (Figures S1–S6). The API-OSs were stored at −4 °C until further use.

### 3.2. Simultaneous Thermogravimetry Analysis

The thermal stability of the compounds was evaluated using a Hitachi STA7200RV instrument (Scancsi, Vila Nova de Gaia, Portugal), following the manufacturer’s instructions. The compounds were subjected to heating from room temperature to 500 °C at a speed of 5 °C/min, obtaining the thermograms provided in Figure 2. For better visualization of the degradative events, the derivatives (red trace) of the thermogravimetric curves (blue trace) are also displayed in the thermograms.

### 3.3. Bacteria

*Mycobacterium avium* 2447 smooth transparent (SmT) strain (kindly provided by Dr. F. Portaels, Institute of Tropical Medicine, Antwerp, Belgium) was grown to the mid-log phase in Middlebrook 7H9 medium (BD Difco^TM^, Fisher Scientific, Porto Salvo, Portugal) containing 0.2% of glycerol (VWR International, Carnaxide, Portugal) and 10% of Albumin-Dextrose-Catalase (ADC, Merck KGaA, Darmstadt, Germany) supplement at 37 °C, as described previously [40].

### 3.4. Direct Effect of Test Compounds against Mycobacteria

The antimicrobial activity of the different compounds was assessed by broth microdilution, following the Clinical and Laboratory Standards Institute (CLSI) guidelines [41] and as described previously [40], using stock solutions of the compounds in DMSO, due to the negligible solubility of Clf in water [37]. Briefly, *M. avium* was grown in Middlebrook 7H9 medium to the exponential phase and 1 to 5 × 10^5^ CFU/mL of bacteria was seeded in 96-well plates with increasing concentrations of the compounds. Each condition was tested in triplicate. The plates were incubated at 37 °C in a humid atmosphere. After 6 days of incubation, bacterial viability was assessed by resazurin reduction. Then, 10% (*v*/*v*) of resazurin (Sigma-Aldrich, St. Louis, MO, USA) at 2.5 mM in phosphate-buffered saline (PBS, Sigma-Aldrich, St. Louis, MO, USA) was added to each well, and after 24 h of incubation at 37 °C, the fluorescence of resorufin, resulting from the conversion of resazurin by metabolically active cells, was measured at λ_ex_ = 530 nm and λ_em_ = 590 nm in a Synergy^TM^ Mx microplate reader from BioTek (Agilent Technologies Inc., Santa Clara, CA, USA) using the Gen5 software also from BioTek. The results are expressed as the percentage of the fluorescence obtained in experimental wells relative to the fluorescence obtained in nontreated wells.

### 3.5. Bone Marrow-Derived Macrophages (BMM)

Macrophages were derived from the bone marrow of male C57BL/6 mice bred at the i3S animal facility and infected with *M. avium* 2447 SmT as described previously [42]. Immediately after infection, BMM were treated with different concentrations of each compound. Each condition was tested in triplicate. The intracellular growth of *M. avium* 2447 SmT was evaluated 5 days after infection, by colony-forming units (CFUs) assay, plating the bacteria in Middlebrook 7H10 agar (BD DifcoTM, Fisher Scientific, Porto Salvo, Portugal) supplemented with 10% Oleic Acid-Albumin-Dextrose-Catalase (OADC; Merck KGaA, Darmstadt, Germany) for at least 7 days at 37 °C. The viability of macrophages was determined by resazurin reduction as detailed above for mycobacteria.

### 3.6. IC_50_ and LD_50_ Determination

The viability of both mycobacteria and host macrophages was determined by resazurin reduction as detailed above. IC_50_ (concentration that inhibits 50% of mycobacterial growth after 7 days of incubation) and LD_50_ (concentration that inhibits 50% of macrophage viability) values were interpolated by fitting the experimental data through a 4PL nonlinear sigmoidal curve using the GraphPad Prism software version 9 (GraphPad Software Inc., La Jolla, CA, USA). The respective 95% confidence interval of the interpolations was also retrieved.

## 4. Conclusions

Two new Clf-derived OSs were produced by a simple acid–base reaction between this drug and two fluoroquinolones, namely Of and Nf, in near-quantitative yields. ^1^H-NMR analysis demonstrated that a 1:1 cation:anion proportion was present in both cases, with complete transfer of the acidic proton from the fluoroquinolone to Clf. Both API-OSs showed good thermal stability, as determined by STA, and improved solubility over Clf, thus reducing one of the major disadvantages of this drug. This was achieved without either substantial loss of antimycobacterial action or increase in toxicity to host cells, compared to the parent antimycobacterial drug. This is unprecedented, as despite there being previous reports addressing the combination of Clf with bioactive carboxylic acids such as p-aminobenzoic [43] and p-aminosalicylic acid [44], the antimycobacterial activity and cytotoxicity of the resulting OSs were not assessed, hence their therapeutic potential remains to be demonstrated.

Diverse formulation strategies have been investigated to overcome oral bioavailability limitations of Clf arising from its poor solubility in aqueous/polar environments: encapsulation in different types of nanoparticles [45,46], nanosuspensions [47], emulsions [48], and other organic salts [49]. This is clear evidence that any gain in Clf solubility is a plus towards the medical application of this drug, highlighting the relevance of research efforts in such direction. This is the case of the present study that paves the way towards new cost-effective strategies to improve the therapeutic index of Clf. It could be argued that some ILs and OSs are quite expensive, but their cost is highly variable, depending on the specific ions that compose them and their respective sources [50]; for instance, quite affordable personal care products can be found in supermarkets shelves that include cetyl pyridinium chloride, a widely known IL used as an active antiseptic ingredient in such products and recently reported to have virucidal (including on SARS-CoV-2) activity [51]. In the specific case of our work, the OSs are obtained by simply mixing two existing APIs, Clf and a fluoroquinolone, and this is undeniably a much simpler and more cost-effective way to ameliorate Clf than most of the nanoformulation strategies mentioned above.

The therapeutic relevance of Clf-fluoroquinolone ionic conjugates as those herein reported may go beyond leprosy treatment, as Clf has been repurposed for inclusion in combination therapies to tackle multidrug-resistant tuberculosis [52] and is known to be active against non-Mycobacteria pathogens, e.g., *Staphylococcus* sp., *Streptomyces* sp., *Listeria* sp., and other bacilli [36]. Therefore, using bactericidal agents such as fluoroquinolones as the counter-ions in Clf-derived OSs may become an effective way to afford a double therapeutic benefit, by boosting not only the antimycobacterial but also the general antibacterial action of Clf, including against opportunistic pathogens that colonize leprous lesions [53]. Ongoing assays will allow a full assessment of the new Clf-derived OSs herein reported, namely testing the compounds in more complex in vitro models of infection and assessing their in vivo antimycobacterial activity. This will hopefully consolidate the value of these API-OSs as a simple and affordable way to ameliorate the physico-chemical profile of Clf without putting its bioactivity at stake or even leading to its improvement.

## Figures and Tables

**Table 1:** Physico-chemical properties and in vitro effects of the API-OSs and their parent drugs. The compounds were tested against both axenic cultures of *M. avium* 2447 SmT (IC_50_) and mammalian host cells (bone marrow-derived macrophages, BMM, LC_50_) infected with *M. avium* 2447 SmT. ^1^.

Compound	Molecular Weight/g·mol^−1^	Net Charge ^2^	Melting Point (Decomposition Temperature)/°C	Solubility/mg·mL^−1^ (mM)	IC_50_/µM ^3^	LC_50_/µM ^4^
Clf	472.12	+1	221.9 (258.0)	4.8 (10.2)	0.33 (0.38 to 0.31) ^5^	4.1 (4.9 to 3.7) ^5^
Of	361.37	−1	274.8 (318.8)	5.7 (15.7)	3.85 (4.78 to 3.20) ^5^	>100
Nf	319.33	−1	206.1 (222.0)	11.0 (34.4)	7.93 (10.94 to 6.25) ^5^	>100
[Clf][Of]	833.26	0	212.0 (274.3)	13.9 (15.8)	0.34 (0.36 to 0.32) ^5^	6.7 (13.04 to 3.42) ^5^
[Clf][Nf]	791.25	0	202.0 (354.6)	13.0 (17.3)	0.35 (0.38 to 0.33) ^5^	6.9 (13.44 to 3.57) ^5^

^1^ Due to the negligible solubility of Clf in water, stock solutions of all compounds were prepared in dimethylsulfoxide, so solubility data refer to this solvent (see text); ^2^ at physiological pH 7.4; ^3^ IC_50_ is the concentration of the compound that inhibits by 50% the mycobacterial viability in axenic culture, obtained by interpolation of nonlinear regression (4PL) of the experimental data; ^4^ LC_50_ is the concentration of the compound that inhibits by 50% the viability of the host cells, obtained by interpolation of nonlinear regression (4PL) of the experimental data; ^5^ the values within the parenthesis represent the 95% confidence interval of the interpolation.

## Data Availability

The data presented in this study are available on request from the corresponding author.

## Supplementary Materials

The following supporting information can be downloaded at: https://www.mdpi.com/article/10.3390/ijms24021402/s1.
