# Supplementary material for: Improving the Antimycobacterial Drug Clofazimine through Formation of Organic Salts by Combination with Fluoroquinolones
Source: Int J Mol Sci. 2023 Jan 11;24(2):1402. doi: 10.3390/ijms24021402 (PMC9865903; doi:10.3390/ijms24021402)
Supplement: Supplementary file 1 [file ijms-24-01402-s001.zip › ijms-2129675-supplementary/SI Mavium.pdf]

Improving the antimycobacterial drug clofazimine through formation of ionic liquids by combination with fluoroquinolones

Clara M. Bento[1,2,3], Ana Teresa Silva[4], Bruno Mansano[1,4], Luísa Aguiar[41], Cátia Teixeira[41], Maria Salomé Gomes[1,6*] Paula Gomes[4], Tânia Silva[1,6], Ricardo Ferraz[4,5*]

1 i3S—Instituto de Investigação e Inovação em Saúde, Universidade do Porto, clara.bento@i3s.up.pt, tania.silva@ibmc.up.pt, bmansano@i3s.up.pt, sgomes@ibmc.up.pt
2 IBMC—Instituto de Biologia Molecular e Celular, Universidade do Porto
3 Programa Doutoral em Biologia Molecular e Celular (MCBiology), Instituto de Ciências Biomédicas Abel Salazar, Universidade do Porto, 4050-313 Porto, Portugal
4 LAQV-REQUIMTE, Departamento de Química e Bioquímica, Faculdade de Ciências; up201303026@edu.fc.up.pt, luisaaguiarts@gmail.com, ricardoferraz@eu.ipp.pt, ca.teixeira@gmail.com, pgomes@fc.up.pt
5 CISA – Ciências Químicas e das Biomoléculas, Escola Superior de Saúde, Politécnico do Porto,
6 ICBAS—Instituto de Ciências Biomédicas Abel Salazar, Universidade do Porto

* Correspondence: sgomes@ibmc.up.pt (MSG); ricardoferraz@eu.ipp.pt (RF)

1. Spectral Data 2
1.1 [Clf][Nf] 2
1.2 [Clf][Of] 5

[1] *Current affiliation*: Gyros Protein Technologies, Tucson, AZ 85714, United States of America

1. Spectral Data

1.1 [Clf][Nf]

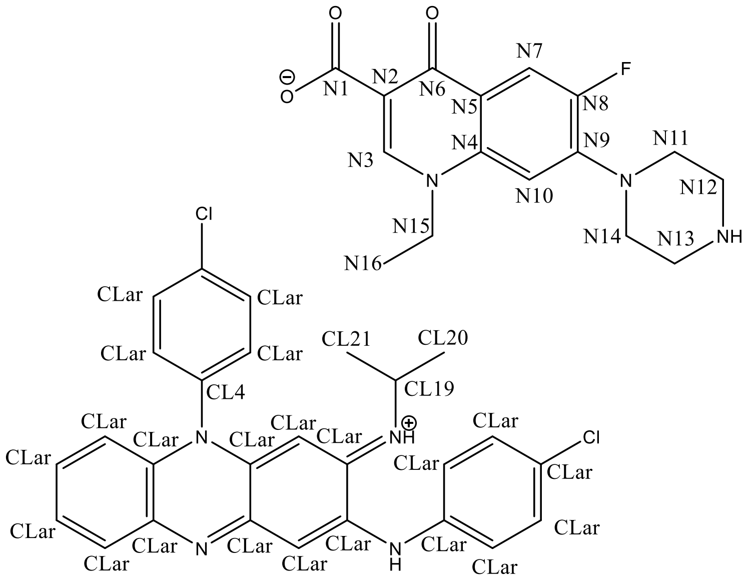

[Clf][Nf]; δH (DMSO-d6, 400 MHz) 8.94(s, 1H,N3), 7.88 (m, 3H, N7 and CLar), 7.61(m, 3H, CLar), 7.44 (m, 4H,CLar), 7.20 (m, 3H, CLar and N10), 6.72 (s, 1H,CLar), 6.45 (m, 1H, CL19), 5.20 (s, 1H,CLar), 4.58 (m, 1H, N15) , 3.23 (m, 4H, N11 and N14), 3.89 (m, 4H, N12 and N13), 1.42(t, 3H, J= 7.1Hz, N16), 1.05 (d, 6H, *J*= 6.2Hz, CL20 and CL21). **δC (DMSO-d6, 100 MHz)** 176.16 (N6), 166.13 (N1), 150.14 (CLar), 150.03 (CLar), 148.44 (N8), 146.10 (N3), 146.00 (N9), 142.96(CL4), 138.87 (CLar), 137.26(N4), 135.87 (CLar) 135.24 (CLar), 134.43 (CLar), 131.57 (CLar), 130.90 (CLar), 129.26 (CLar), 127.80 (CLar), 127.80 (CLar), 122.83 (CLar), 114.05 (CLar), 111.23 (N5), 111.00 (N7), 107.04 (N2), 105.56(N10), 98.93 (CLar), 88.28(CLar), 50.82 (N14), 49.02 (N11), 48.89 (CLar), 45.39 (N12 and N13), 23.34 (CL20 and CL21), 14.30 (N16). **ESI-IT MS (+)** ($C_{27}H_{23}Cl_2N_4^+$, 472.12 u.m.a.) m/z: 473.33 u.m.a. (MH+).

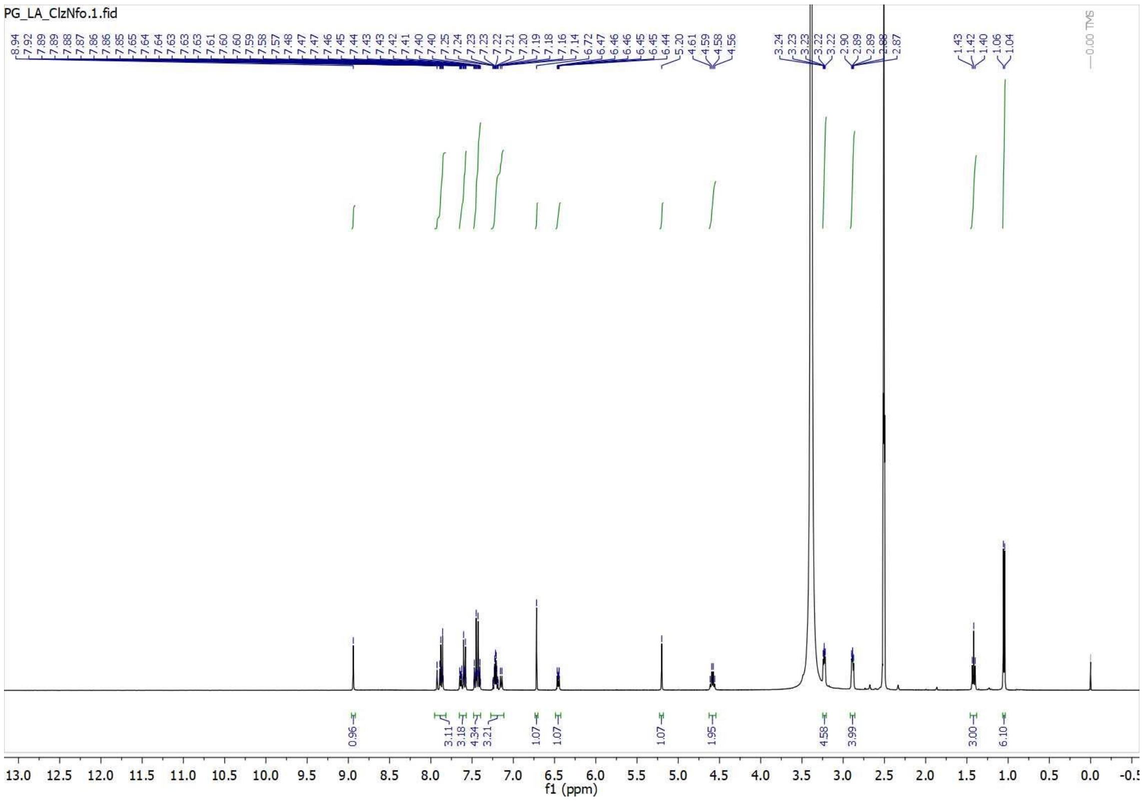

Figure S1. ^{1}H-NMR spectrum of [Clf][Nf] (400 MHz, DMSO-d6).

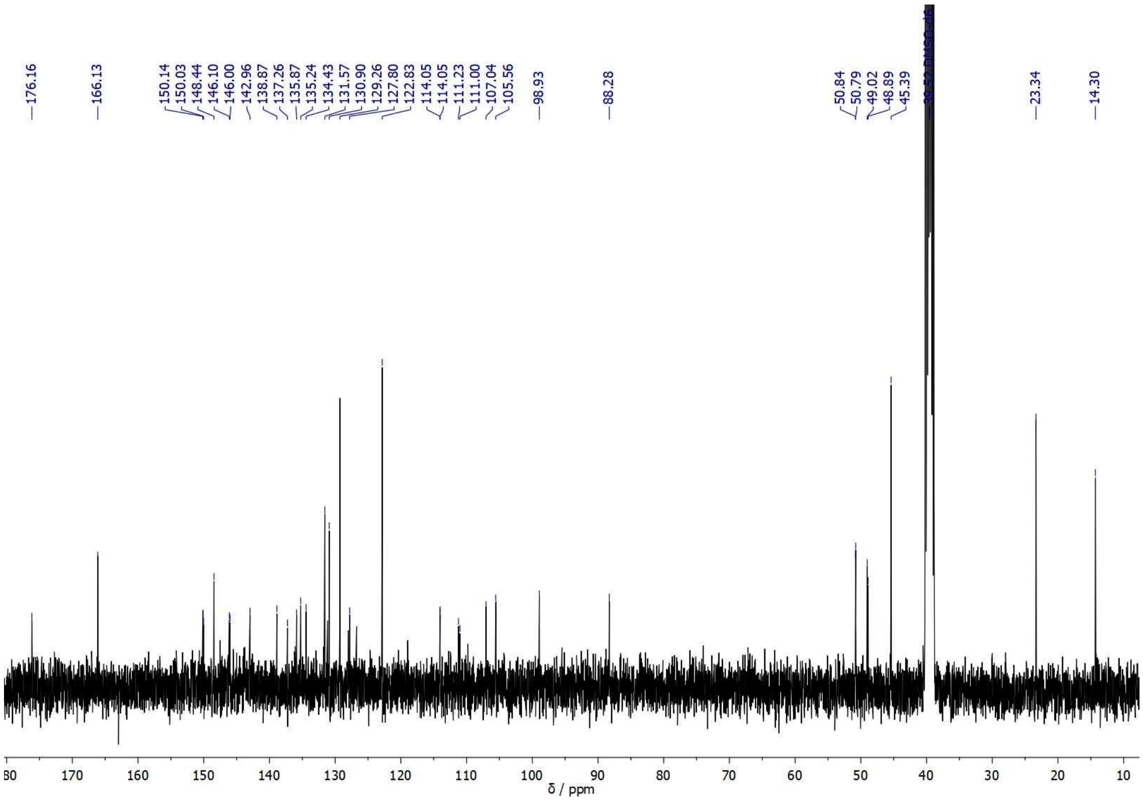

Figure S2. ^{13}C-NMR spectrum of [Clf][Nf] (100 MHz, DMSO-d_6).

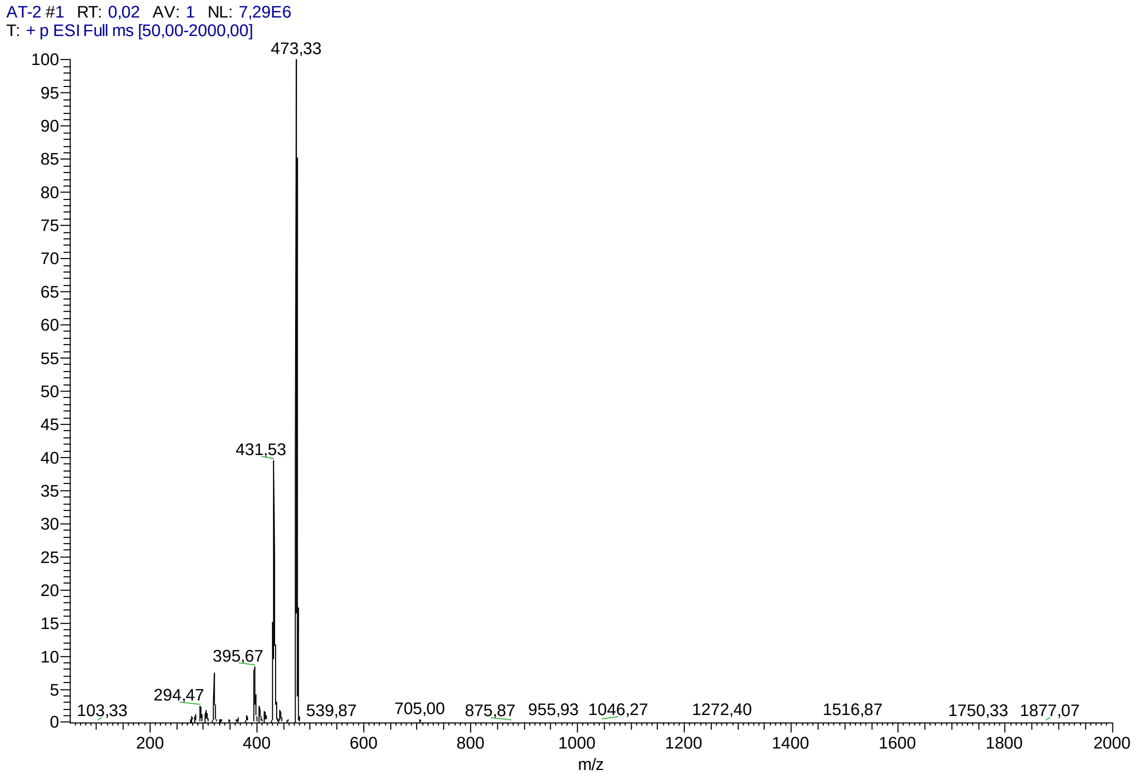

Figure S3. ESI-IT mass spectrum for [Clf][Nf] (positive mode).

1.2 [Clf][Of]

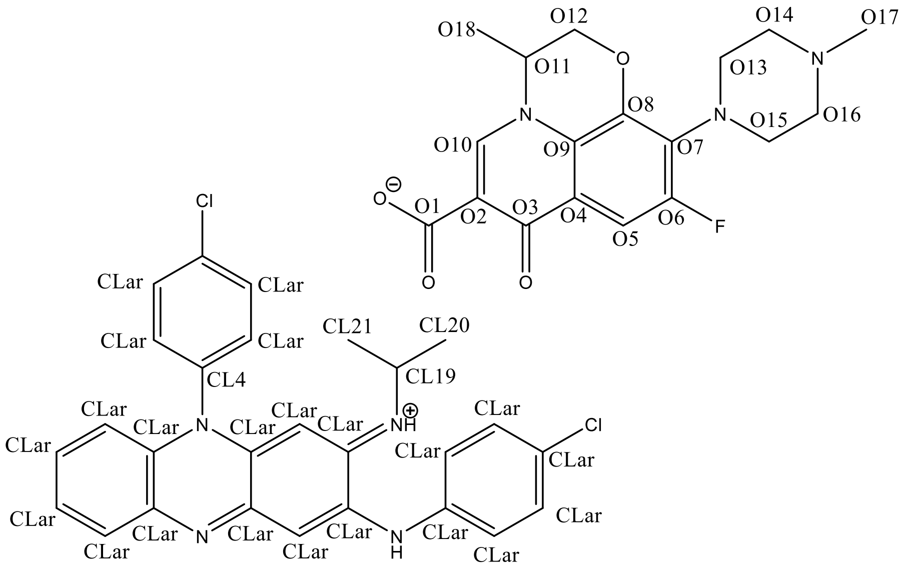

[Clf][Of]; δH (DMSO-d6, 400 MHz) 8.94(s, 1H,O10), 7.87 (m, 2H, CLar), 7.61(m, 4H, CLar and O5), 7.44 (m, 4H,CLar), 7.21 (m, 2H, CLar), 6.72 (s, 1H,CLar), 6.46 (m, 1H, CLar), 5.20 (s, 1H,CLar), 4.89 (m, 1H, O11), 4.57 (m, 1H, O12), 4.37(m, 1H, O12), 3.29 (m, 4H, O13 and O15), 2.44 (m, 4H, O14 and O16), 2.23(s, 3H, O17), 1.45 (d, 3H, *J*= 6.8 Hz, O18) 1.05 (d, 6H, J= 6.2Hz, CL20 and CL21). **δC (DMSO-d6, 100 MHz)** 166.05 (O3), 157.20 (O6), 150.03 (CLar), 142.97 (CL4), 140.21 (O7), 138.87 (CLar), 135.87 (CLar) 135.24 (CLar), 134.43 (CLar), 131.57 (CLar), 130.15(CLar), 130.90 (CLar), 129.26 (CLar), 122.84 (CLar), 119.78 (O2), 114.05 (CLar), 98.94 (CLar), 88.29(CLar), 55.27 (O13 and O15), 50.11 (O14), 48.90 (CL19), 46.03 (O16), 23.34 (CL19 and CL20), 17.88 (O18). **ESI-IT MS (+)** ($C_{27}H_{23}Cl_2N_4^+$, 472.12 u.m.a.) m/z: 473.40 u.m.a. (MH+).

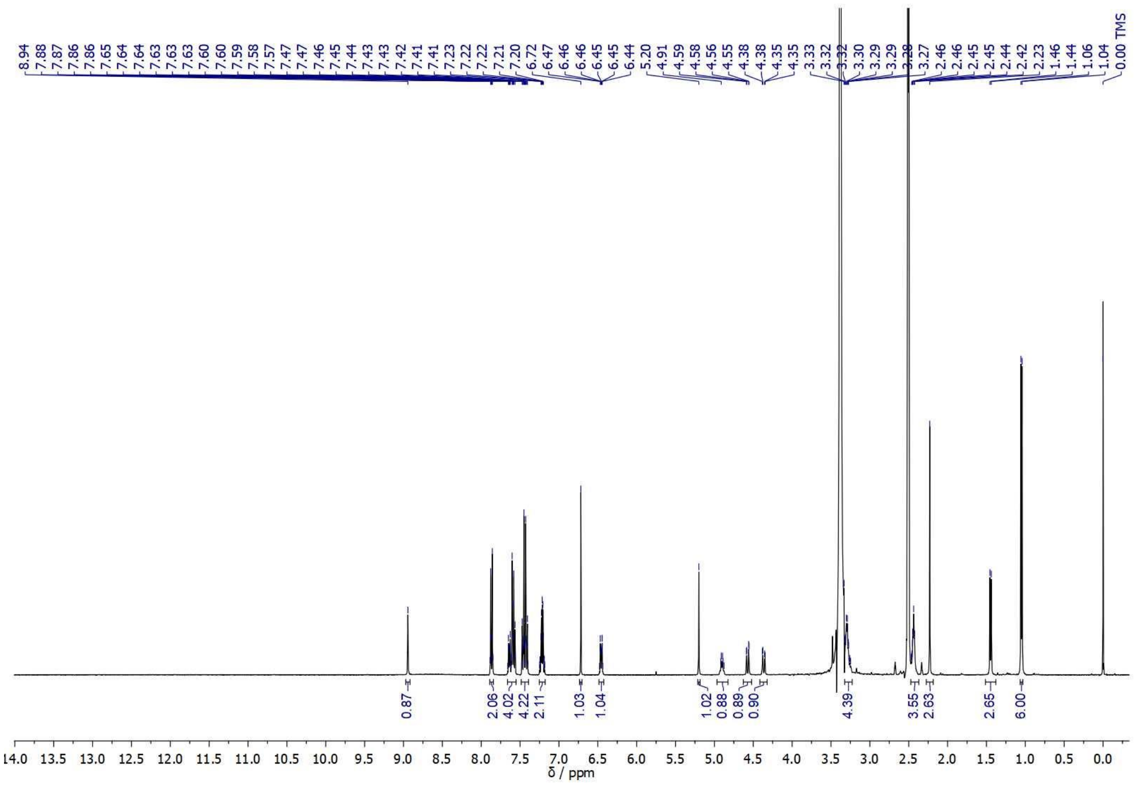

Figure S4. ^{1}H-NMR spectrum of [Clf][Of] (400 MHz, DMSO-d6).

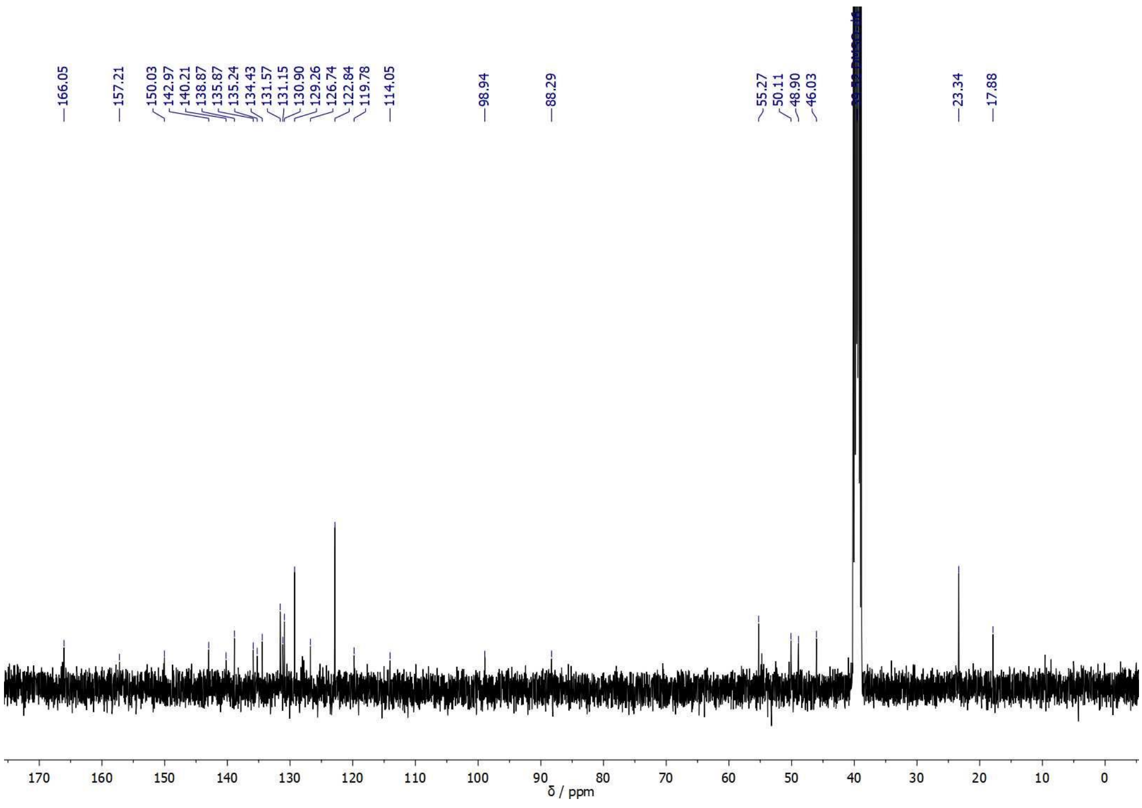

Figure S5. ^{13}C-NMR spectrum of [Clf][Of] (100 MHz, DMSO-d_6).

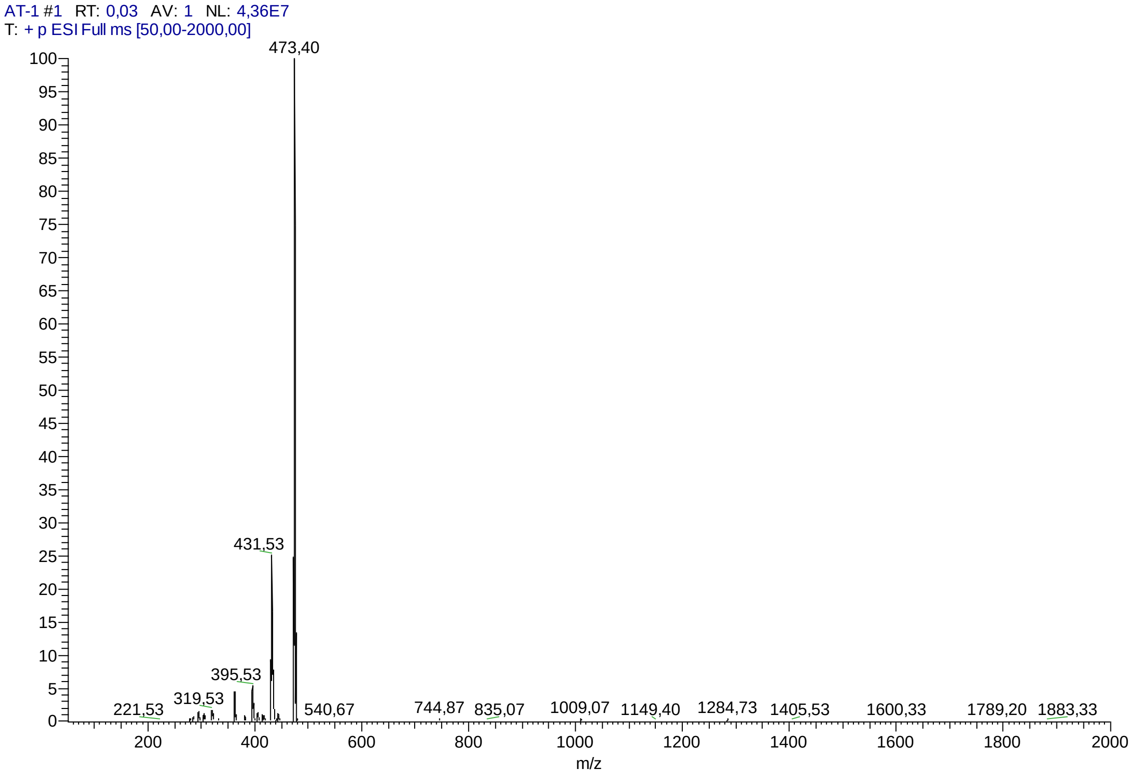

Figure S6. ESI-IT mass spectrum for [Clf][Of] (positive mode).
